# Scaffold-based and calcium-silicate biomaterials in vital pulp therapy: a systematic review of current evidence and translational perspectives

**DOI:** 10.1038/s41405-026-00480-7

**Published:** 2026-10-31

**Authors:** Marisa Menezes Santana, Simon John Barnwell

**Affiliations:** 1 Simplyendo®, Formby, UK; 2 University of Chester, Chester, UK

**Keywords:** Dental biomaterials, Pulp conservation, Root canal treatment

## Abstract

**Objective:**

To critically synthesise and appraise the current evidence on scaffold-based regenerative biomaterials and calcium-silicate biomaterials used in vital pulp therapy (VPT), with particular emphasis on the translational challenges associated with achieving biological regeneration of the dentine–pulp complex.

**Methods:**

This systematic review was conducted in accordance with PRISMA 2020 guidelines. Electronic searches of MEDLINE (via PubMed), Scopus and Web of Science were performed from database inception to March 2025. Studies evaluating scaffold-based regenerative biomaterials or calcium-silicate biomaterials in VPT were included. Human clinical investigations and in vivo animal studies were considered to capture evidence across different stages of translational research. Methodological quality was assessed using study-design-appropriate risk-of-bias tools. Owing to substantial heterogeneity in study design, interventions and outcome measures, findings were synthesised narratively.

**Results:**

Eleven studies met the inclusion criteria, comprising six clinical investigations evaluating calcium-silicate biomaterials and five preclinical animal studies investigating scaffold-based regenerative approaches. Clinical evidence consistently demonstrated favourable outcomes for calcium-silicate biomaterials in preserving pulpal vitality and promoting reparative dentine formation. Scaffold-based systems demonstrated promising regenerative characteristics, including enhanced angiogenesis, odontogenic differentiation and formation of organised pulp-like tissue under experimental conditions. However, scaffold evidence was limited to preclinical models, and no included study directly compared scaffold-based and calcium-silicate biomaterials within the same experimental or clinical framework.

**Conclusions:**

Current evidence supports calcium-silicate biomaterials as the clinical standard for VPT, whereas scaffold-based regenerative biomaterials remain a promising area of translational research. Although experimental findings suggest considerable regenerative potential, the absence of direct comparative clinical evidence precludes conclusions regarding comparative effectiveness. Future well-designed clinical studies are required to determine whether scaffold-based approaches can successfully translate into predictable clinical regeneration of the dentine–pulp complex.

## Introduction

Preservation of the dentine–pulp complex remains a central objective of contemporary endodontic practice. The biological integrity of the pulp is fundamental not only to tooth vitality but also to long-term structural resilience, immunological defence, and proprioceptive function. As a dynamic and highly specialised tissue system, the dentine–pulp complex possesses an intrinsic capacity to respond to injury through coordinated inflammatory and reparative processes aimed at maintaining function [1, 2]. This responsiveness underpins the rationale for increasingly conservative treatment approaches and reflects a broader shift in modern dentistry towards minimally invasive strategies that prioritise tissue preservation.

Encased within rigid dentinal walls, the pulp is a richly vascularised and innervated connective tissue that is particularly susceptible to pressure changes and inflammatory insult. In response to caries, trauma, or operative intervention, it initiates a coordinated cascade involving immune cell recruitment, cytokine signalling, and activation of progenitor cells [1]. These responses are tightly regulated and may culminate in resolution and repair or progress towards irreversible damage, depending on the magnitude and duration of the insult. Importantly, pulpal healing occurs along a continuum rather than as a binary process, influenced by bacterial burden, host immune competence, and the local microenvironment within the pulp chamber.

Within this context, VPT has emerged as a biologically driven treatment modality designed to preserve pulpal vitality in teeth affected by caries or trauma [3, 4]. Historically, pulpal disease was frequently managed through complete extirpation; however, advances in diagnostic understanding and biomaterials have enabled a transition towards more conservative strategies. Contemporary VPT procedures, including direct pulp capping, partial pulpotomy, and full pulpotomy, are supported by a growing body of clinical evidence demonstrating favourable outcomes when appropriate case selection and material selection are applied [5–8].

A key determinant of success in VPT lies in the ability to modulate inflammation while preserving a viable population of pulp cells capable of initiating repair. Controlled inflammatory responses may stimulate differentiation of odontoblast-like cells and subsequent deposition of tertiary dentine, forming a protective barrier against further insult [1, 2]. In contrast, sustained or excessive inflammation compromises this reparative capacity and may lead to irreversible tissue breakdown. This balance underscores the importance of both biological understanding and material performance in shaping clinical outcomes.

The introduction of calcium-silicate biomaterials, particularly mineral trioxide aggregate and Biodentine, has been instrumental in advancing VPT. These materials combine biocompatibility, bioactivity, and effective sealing ability, creating an environment conducive to pulpal healing and hard tissue formation [9, 10]. Their capacity to release calcium ions and promote mineralised interface formation supports cellular differentiation and reparative dentinogenesis, while their sealing properties limit bacterial ingress and subsequent inflammation.

Clinical investigations have consistently reported high success rates associated with these materials across a range of VPT procedures [4–8]. This reliability has established them as the current standard of care. Nevertheless, the biological response they elicit is predominantly reparative rather than regenerative.

Reparative dentinogenesis results in the formation of a dentine bridge that, although clinically protective, does not fully replicate the structural or functional characteristics of native dentine [11]. The resulting tissue often exhibits irregular tubular organisation, reduced mechanical properties, and limited integration with the existing dentine–pulp complex. From a biological perspective, this represents healing rather than true regeneration. Complete regeneration would require restoration of native architecture, including organised odontoblast layers, vascular networks, and neural components [11–13].

These limitations have driven increasing interest in regenerative endodontics, an evolving field focused on restoring functional pulp tissue through biologically driven approaches [12, 14–17]. Grounded in tissue engineering principles, these strategies aim to harness interactions between scaffolds, signalling molecules, and stem or progenitor cells to promote organised tissue formation and functional recovery.

Among these approaches, regenerative scaffold systems have attracted particular attention. Biomimetic constructs provide a three-dimensional framework that facilitates cellular attachment, proliferation, and differentiation while supporting vascularisation and nutrient diffusion [13, 18]. Advances in biomaterial engineering, including hydrogels and three-dimensional printed matrices, have enabled increasingly sophisticated designs that more closely replicate the native extracellular environment. Beyond their structural role, such systems may also function as delivery platforms for bioactive molecules, including growth factors and signalling peptides, thereby enhancing cellular recruitment and guiding tissue development within the pulp space. This integration of structural and biochemical cues represents a critical step towards achieving functional regeneration rather than mere tissue replacement [19].

Experimental studies have reported promising outcomes, including the formation of vascularised pulp-like tissue, deposition of dentine-like structures, and expression of odontogenic markers [20–24]. These findings suggest that regenerative scaffold approaches may overcome some of the inherent limitations of conventional biomaterials. However, despite these advances, translation into predictable clinical outcomes remains constrained.

Multiple factors contribute to this translational gap. Successful regeneration of the dentine–pulp complex requires precise coordination of vascularisation, cellular signalling, and host–material interactions. The establishment of a functional vascular network is particularly critical, as sustained tissue viability depends on adequate perfusion. In addition, host immune response and the multifactorial nature of the in vivo environment introduce variables that are difficult to replicate under controlled experimental conditions [1, 11, 13, 16].

Consequently, while regenerative scaffold systems reveal considerable potential in experimental settings, their clinical applicability remains uncertain. In contrast, calcium-silicate biomaterials continue to deliver predictable and reproducible outcomes within a reparative framework.

This divergence between experimental promise and clinical reliability underscores a persistent translational gap in the current evidence base. A structured evaluation of the available literature is therefore required to critically examine the current evidence supporting these biomaterial strategies and to identify the translational challenges that continue to limit clinical regeneration.

Accordingly, this systematic review aimed to critically synthesise the current evidence relating to scaffold-based regenerative biomaterials and calcium-silicate biomaterials in VPT. Emphasis was placed on identifying the regenerative potential, clinical performance and methodological limitations of the available evidence, while exploring the translational challenges that currently limit the progression of scaffold-based regenerative strategies from experimental models to routine clinical practice.

## Methods

### Registration and reporting

This systematic review was conducted according to a predefined methodology established prior to the initiation of the literature search and is reported in accordance with the Preferred Reporting Items for Systematic Reviews and Meta-Analyses (PRISMA 2020) statement [25]. The review protocol was not prospectively registered (e.g. PROSPERO), as the study was undertaken within an academic research framework. Nevertheless, the review question, eligibility criteria, search strategy, and planned methods of synthesis were established a priori and remained unchanged throughout the review. The absence of prospective protocol registration is acknowledged as a methodological limitation and was considered when interpreting the findings.

### Focus question and PICO framework

The review sought to answer the following focus question:

**“**What does the current evidence reveal about scaffold-based regenerative biomaterials and calcium-silicate biomaterials in vital pulp therapy, and what are the implications for their translation into clinical practice?”

The review question was developed using the Population, Intervention, Comparison, and Outcome (PICO) framework to ensure a structured and transparent approach to study selection.

- Population (P): Teeth undergoing VPT.
- Intervention (I): Scaffold-based regenerative biomaterials designed to support regeneration of the dentine–pulp complex.
- Comparison (C): Calcium-silicate biomaterials used in VPT, including mineral trioxide aggregate (MTA) and Biodentine.
- Outcome (O): Biological, histological, radiographic and clinical outcomes relating to regeneration or preservation of the dentine–pulp complex, including maintenance of pulpal vitality, tissue organisation and dentine formation.

### Search strategy

A comprehensive electronic search of the published literature was undertaken in MEDLINE (accessed via PubMed), Scopus and Web of Science from database inception to March 2025 to identify studies evaluating scaffold-based regenerative biomaterials and calcium-silicate biomaterials in VPT.

The electronic search strategy combined controlled vocabulary (Medical Subject Headings [MeSH], where applicable) with free-text keywords relating to VPT, scaffold-based regenerative biomaterials and calcium-silicate biomaterials. Boolean operators (AND/OR) were used to combine search concepts and maximise retrieval of relevant studies while maintaining search specificity.

The core search strategy included the terms *“vital pulp therapy”*, *“pulpotomy”*, *“direct pulp capping”*, *“scaffold”*, *“hydrogel”*, *“3D printed scaffold”*, *“tissue engineering”*, *“calcium silicate”*, *“mineral trioxide aggregate”* and *“Biodentine”*. The complete electronic search strategy for MEDLINE (via PubMed) is provided in Supplementary Material 1. Equivalent search strategies were adapted for Scopus and Web of Science using database-specific syntax.

Retrieved records were screened according to the predefined eligibility criteria. Only articles published in English were considered for inclusion. Eligible study designs comprised human clinical investigations and in vivo animal studies. In addition to electronic database searching, the reference lists of all included studies and relevant review articles were manually screened to identify any additional eligible publications.

### Eligibility criteria

Eligibility criteria were established a priori in accordance with the review question and PICO framework to ensure a transparent, reproducible, and consistent study selection process.

Studies were considered eligible for inclusion if they satisfied all the following predefined eligibility criteria:

### Inclusion criteria

- *Population*: Teeth undergoing VPT.
- *Intervention*: Scaffold-based regenerative biomaterials or calcium-silicate biomaterials used in VPT.
- *Study design*: Human clinical studies and in vivo animal investigations.
- *Outcomes*: Biological, histological, radiographic and/or clinical outcomes relating to preservation or regeneration of the dentine–pulp complex.
- *Publication characteristics*: Full-text articles published in English.

### Exclusion criteria

Studies were excluded if they met one or more of the following criteria:

- were narrative reviews or expert opinion articles.
- were in vitro studies without in vivo validation.
- were not related to VPT or pulp regeneration.
- did not evaluate scaffold-based regenerative biomaterials or calcium-silicate biomaterials.
- did not report biological or clinical outcomes relevant to preservation or regeneration of the dentine–pulp complex.

### Study selection and data extraction

All records identified through the electronic database searches were imported into EndNote, where duplicate records were identified and removed prior to screening.

Titles and abstracts of all retrieved records were screened against the predefined eligibility criteria by one reviewer (M.S.). Studies considered potentially eligible subsequently underwent full-text assessment by the same reviewer. Study selection and eligibility decisions were verified by a second reviewer (S.B.), with any uncertainties resolved through discussion and consensus.

Data extraction was performed by one reviewer (M.S.) using a standardised data extraction form developed specifically for this review. The following data were extracted from each included study: study design, experimental model or clinical population, sample size, biomaterial or scaffold composition, intervention characteristics, outcome measures, duration of follow-up, and principal findings. The extracted data were subsequently verified by a second reviewer (S.B.) to ensure accuracy and completeness. Where multiple publications reported the same study population, the most recent publication was used for data extraction to minimise duplication of data.

### Quality appraisal

The methodological quality of the included studies was assessed using appraisal tools appropriate to the study design. The SYRCLE Risk of Bias tool was used to evaluate in vivo animal studies, while the Joanna Briggs Institute (JBI) Critical Appraisal Tools were used to assess human clinical studies [26, 27].

Methodological quality assessment was undertaken by one reviewer (M.S.**)** using the predefined criteria for each appraisal tool. The completed assessments were subsequently reviewed by a second reviewer **(**S.B.**)**, with any uncertainties resolved through discussion and consensus.

No studies were excluded on the basis of methodological quality or risk of bias. Instead, the findings of the quality appraisal informed the interpretation of the evidence and the overall conclusions of the review.

### Certainty of evidence

Given the substantial clinical and methodological heterogeneity across the included studies, together with the absence of a quantitative synthesis, a formal GRADE assessment was not undertaken. Instead, the overall certainty of the evidence was considered narratively, taking into account study limitations, risk of bias, indirectness of the evidence, inconsistency across studies, and imprecision, consistent with published guidance for assessing certainty of evidence in systematic reviews without a single pooled estimate of effect [28].

### Data synthesis

A quantitative meta-analysis was not undertaken because of substantial clinical and methodological heterogeneity across the included studies. Heterogeneity was evident in study design, experimental models, biomaterial composition, intervention protocols, outcome measures, and follow-up periods, precluding meaningful quantitative pooling of the data.

Instead, the findings were synthesised narratively using a structured approach. Studies were grouped according to biomaterial category (scaffold-based regenerative biomaterials and calcium-silicate biomaterials), study design and reported outcomes to facilitate comparison of the available evidence. The narrative synthesis was undertaken to identify patterns in biological response, regenerative potential and clinical outcomes while considering methodological quality, risk of bias, and the overall certainty of the evidence during interpretation of the findings.

### Ethics approval and consent to participate

Ethical approval was not required as this study involved analysis of previously published data.

## Results

The database search identified 1240 records. Following removal of 621 duplicate records, 619 studies underwent title and abstract screening. Of these, 30 articles were assessed for full-text eligibility, of which 11 met the predefined inclusion criteria and were included in the qualitative synthesis.

**Fig. 1:**
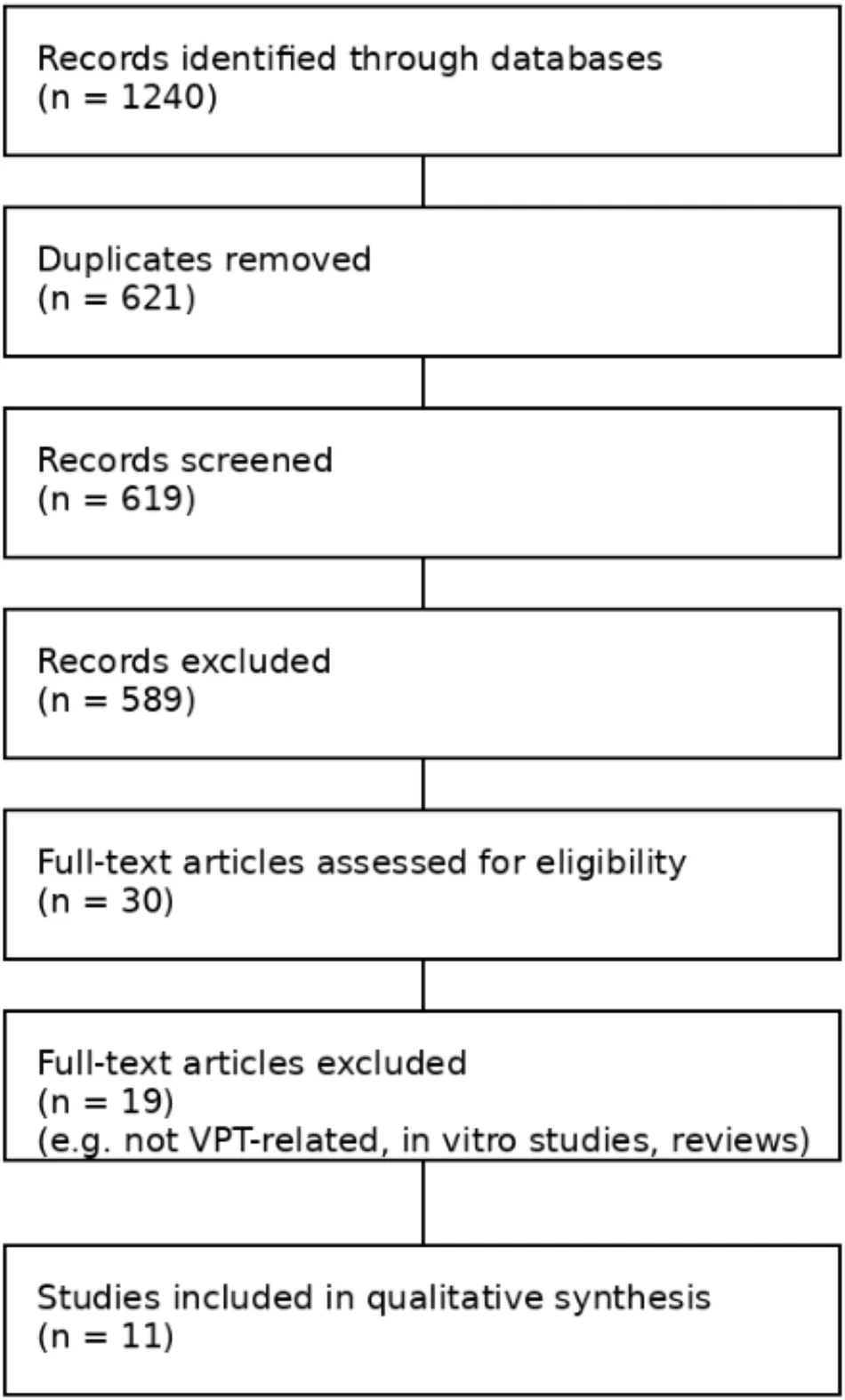
PRISMA 2020 study selection flow diagram. Flow diagram illustrating the identification, screening, eligibility assessment and inclusion of studies in the qualitative synthesis according to the PRISMA 2020 reporting guidelines.

The included studies comprised five randomised clinical trials, one prospective cohort study, and five in vivo animal investigations. The study selection process is illustrated in Fig. 1, and the characteristics of the included studies are summarised in Table 1.

**Table 1:** Characteristics of the Included Studies

Author (Year)	Study type	Model/population	Intervention (scaffold/material)	Comparator	Outcome measures	Key findings	Sample size	Follow-up	Risk of bias	Study limitations
Cunha et al. (2023)	Animal	Rat molars	GelMA microgel scaffold + dentine matrix proteins	Control	Angiogenesis, tissue formation	Increased vascularisation and pulp-like tissue formation	Not clearly reported	Short-term	Moderate	No microbial challenge; limited reporting of randomisation/blinding
Han et al. (2024)	Animal	Rat model	GelMA/β-TCP nanocomposite scaffold	Control	Odontogenic differentiation	Enhanced expression of odontogenic markers	Not clearly reported	Short-term	Moderate	Limited methodological reporting; preclinical model
Tao et al. (2025)	Animal	In vivo model	Injectable bioceramic hydrogel scaffold	Control	Neurovascular regeneration	Improved neurovascular integration and tissue organisation	Not clearly reported	Short-term	Moderate	No microbial challenge; limited translational relevance
Yi et al. (2024)	Animal	Rat model	Hierarchical hydrogel scaffold	Control	Tissue organisation	Formation of structured pulp-like tissue	Not clearly reported	Short-term	Moderate	Preclinical setting; lack of long-term validation
Zhang et al. (2024)	Animal	Rabbit model	Nanocomposite scaffold	Control	Regenerative outcomes	Enhanced dentine–pulp complex regeneration	Not clearly reported	Short-term	Moderate	Species differences; absence of clinical conditions
Aguilar et al. (2021)	Clinical (RCT)	Human permanent teeth	MTA / Biodentine	Comparison	Vitality, dentine bridge formation	High clinical success and predictable outcomes	Moderate	12–24 months	Low–Moderate	Limited blinding; variability in case selection
Silva et al. (2025)	Clinical (RCT)	Human permanent teeth	Biodentine pulpotomy	Root canal treatment	Clinical outcomes	Comparable outcomes to root canal treatment	Moderate	12–24 months	Moderate	Sample size limitations; follow-up variability
Taha et al. (2023)	Clinical (RCT)	Human permanent teeth	Bioceramic putty pulpotomy	Root canal treatment	Vitality preservation	High success rates at follow-up	Moderate	24 months	Low–Moderate	Operator variability; limited blinding
Zhu et al. (2024)	Clinical (cohort)	Human permanent teeth	iRoot BP Plus	Root canal treatment	Clinical outcomes	Positive vitality and healing outcomes	Moderate	12–24 months	Moderate	Non-randomised design; potential selection bias
Baranwal et al. (2025)	Clinical (RCT)	Human permanent teeth	Biodentine vs MTA	Comparison	Clinical success	Comparable performance between materials	Moderate	12 months	Low–Moderate	Short follow-up; limited sample size
Gilhotra et al. (2024)	Clinical (RCT)	Human permanent teeth	Biodentine, MTA, Bio-C Repair, EndoSequence BC-RRM	Comparison	Clinical outcomes	Consistently favourable results across materials	Moderate	12–24 months	Moderate	Heterogeneity in materials and protocols

The included evidence comprised five in vivo animal investigations evaluating scaffold-based regenerative biomaterials and six human clinical studies evaluating calcium-silicate biomaterials. Scaffold-based regenerative biomaterials were investigated exclusively in experimental animal models, whereas calcium-silicate biomaterials were assessed predominantly in permanent human teeth (Table 1).

### Findings from scaffold-based regenerative biomaterials

Scaffold-based regenerative biomaterials were evaluated exclusively in in vivo animal models, including rat and rabbit models (Table 1). Sample sizes were generally small or not consistently reported, and follow-up periods were limited to short-term experimental observation.

Reported outcomes included increased angiogenesis, upregulation of odontogenic markers, and the formation of organised pulp-like tissue structures [20–24] In several studies, vascularised tissue resembling native pulp was observed alongside deposition of dentine-like structures [20–24] Scaffold interventions varied considerably in composition, architecture, and delivery systems, including hydrogel-based constructs, nanocomposite scaffolds, injectable bioceramic hydrogels, and three-dimensional printed scaffold systems (Table 1) [20–24].

### Findings from calcium-silicate biomaterials

Clinical studies evaluating calcium-silicate biomaterials comprised five randomised clinical trials and one prospective cohort study involving permanent human teeth, with moderate sample sizes and follow-up periods typically ranging from 12 to 24 months (Table 1).

Clinical studies consistently reported high rates of pulpal vitality preservation and successful formation of reparative dentine bridges following VPT using calcium-silicate biomaterials, including mineral trioxide aggregate (MTA) and Biodentine [29–34]. Comparable clinical outcomes were also reported in studies evaluating pulpotomy using calcium-silicate biomaterials against root canal treatment [30–32]. Although overall success rates were high, some variability was observed between studies with respect to treatment protocols, follow-up duration, and reported clinical outcomes [29–34].

### Comparative synthesis

No included studies directly compared scaffold-based regenerative biomaterials with calcium-silicate biomaterials within the same experimental or clinical study. Comparisons between regenerative scaffold approaches and calcium-silicate biomaterials were therefore indirect [20–24,29–34]. Effect sizes were not consistently reported across the included studies, precluding quantitative comparison and supporting the decision to undertake a narrative synthesis.

### Risk of bias and methodological quality

Assessment of methodological quality demonstrated variability across both experimental and clinical studies.

Animal studies evaluated using the SYRCLE Risk of Bias tool demonstrated an overall moderate risk of bias, primarily due to limited reporting of randomisation procedures, allocation concealment, and blinding of outcome assessment.

Clinical studies assessed using the Joanna Briggs Institute (JBI) Critical Appraisal Tools generally exhibited moderate to high methodological quality. Identified limitations included moderate sample sizes, variability in follow-up duration (typically 12–24 months), lack of blinding in some studies, operator-dependent variability, and potential selection bias associated with the prospective cohort study design.

## Discussion

The present systematic review highlights a divergence between biological innovation and clinical translation in VPT. While scaffold-based regenerative biomaterials consistently demonstrated biological processes associated with dentine–pulp regeneration under experimental conditions, the current clinical evidence continues to support calcium-silicate biomaterials as the most predictable and reliable treatment option. Rather than representing conflicting findings, these observations reflect two distinct stages of translational development: scaffold-based regenerative biomaterials illustrate what may be biologically achievable, whereas calcium-silicate biomaterials represent what is currently reproducible in clinical practice. Consequently, the principal challenge facing regenerative endodontics is no longer demonstrating biological feasibility but translating these biological advances into predictable and clinically applicable therapies.

Both scaffold-based regenerative biomaterials and calcium-silicate biomaterials seek to achieve the same fundamental clinical objective: preservation of the vitality and function of the dentine–pulp complex. Consequently, considering these biomaterial strategies within the same systematic review provides an opportunity to evaluate the current evidence supporting each approach while recognising their differing stages of translational development. Importantly, the available literature does not permit direct comparison of their relative clinical performance, as scaffold-based regenerative biomaterials have been investigated almost exclusively in preclinical models, whereas calcium-silicate biomaterials have been evaluated predominantly in human clinical studies. Accordingly, comparisons within the present review should be interpreted as contextual evaluations of two biologically relevant treatment strategies rather than direct assessments of comparative clinical effectiveness.

The biological performance demonstrated by scaffold-based regenerative biomaterials across the included preclinical studies warrants careful consideration. Unlike calcium-silicate biomaterials, which primarily support reparative healing by providing a biologically favourable environment, scaffold-based regenerative biomaterials are specifically engineered to influence the regenerative microenvironment itself [12–17]. Across the included animal studies, scaffold systems consistently promoted angiogenesis, enhanced expression of odontogenic markers, and supported the formation of organised pulp-like tissue [20–24]. These findings suggest that scaffold-based regenerative biomaterials have the capacity to recreate important structural and biological features of the native dentine–pulp complex under controlled experimental conditions. Collectively, the available preclinical evidence illustrates the considerable advances achieved in biomaterials science and tissue engineering, while also highlighting the potential of scaffold-based regenerative strategies to extend beyond conventional reparative approaches.

Despite these encouraging biological findings, successful translation into reproducible therapeutic outcomes remains a considerable challenge. Regeneration of the dentine–pulp complex requires far more than the initiation of isolated biological processes; it depends upon the coordinated integration of angiogenesis, cellular differentiation, immune modulation, extracellular matrix remodelling, and functional tissue organisation within a highly constrained biological environment [1, 11–13, 16]. While these processes can be carefully controlled under experimental conditions, they are influenced in clinical practice by numerous variables, including microbial contamination, host immune responses, patient-specific biological variation, and procedural factors. Notably, most preclinical studies included in this review were conducted under controlled experimental conditions without microbial challenge, limiting their ability to replicate the complex biological environment encountered in clinical VPT [20–24]. Consequently, biological success achieved under experimental conditions should not be assumed to translate directly into consistent clinical outcomes.

One possible explanation for the translational paradox identified in the present review is the existence of a biological ceiling within contemporary VPT. This concept should not be interpreted as a fixed biological limit, but rather as an interpretative framework that emerges from the current evidence. Calcium-silicate biomaterials consistently achieve highly reproducible reparative outcomes that preserve pulpal vitality and support long-term clinical success [9, 10, 35–37]. However, despite the increasingly sophisticated biological responses demonstrated by scaffold-based regenerative biomaterials under experimental conditions, convincing evidence that these approaches can reproducibly exceed the clinical outcomes already achieved with calcium-silicate biomaterials remains unavailable [20–24]. Within this context, the biological ceiling may represent the point at which increasing biological sophistication no longer translates proportionately into clinically measurable benefit, at least with the technologies, outcome measures, and evidence currently available. This hypothesis does not imply that biological innovation has reached its limit; rather, it suggests that clinical translation may currently represent the principal barrier to further therapeutic advancement.

The hypothesis of a biological ceiling is biologically plausible when the intrinsic characteristics of the dentine–pulp complex are considered [1, 11, 12]. Unlike many other tissues, the dental pulp exists within a rigid, low-compliance environment in which vascular perfusion, immune regulation, neural function, and dentinogenesis must remain precisely coordinated to maintain tissue homeostasis [13, 16]. Successful regeneration therefore requires considerably more than the initiation of isolated biological processes; it depends upon the spatial and temporal integration of multiple cellular and molecular events within a confined anatomical space. Although scaffold-based regenerative biomaterials have demonstrated the capacity to promote angiogenesis, cellular differentiation, and organised tissue formation under experimental conditions, reproducing the structural organisation and functional integration of native pulp tissue within the clinical environment remains substantially more complex [20–24]. Consequently, increasing biological sophistication may not be sufficient to generate proportionately greater clinical benefit unless these regenerative processes can be translated into consistent, durable, and functionally integrated healing.

The interpretation of these findings must also be considered in the context of the available evidence. The inclusion of both animal and human studies was necessary to capture the continuum of evidence across the translational pathway, from mechanistic investigation to established clinical application. Animal models provide invaluable insight into biological mechanisms that cannot be investigated directly in human clinical studies, particularly with respect to angiogenesis, tissue organisation, and cellular differentiation [1, 11–13, 16]. However, they cannot fully replicate the biological complexity of human disease, including microbial challenge, age-related variation, host immune responses, and the procedural variables encountered in routine clinical practice. Consequently, while preclinical investigations remain essential for understanding regenerative mechanisms, their findings should be interpreted as informing biological plausibility rather than predicting clinical effectiveness.

The consistently favourable clinical performance of calcium-silicate biomaterials is unlikely to be explained by a single biological mechanism. Rather, their success appears to reflect the integration of several complementary properties, including excellent sealing ability, bioactivity, biocompatibility, and favourable antimicrobial characteristics [9, 10]. Together, these properties create a stable biological environment that supports predictable reparative healing, an outcome consistently supported by the available clinical evidence [35–37]. Importantly, successful VPT depends not only upon stimulating tissue repair, but also upon effective control of microbial contamination. In this respect, calcium-silicate biomaterials combine biological activity with clinical practicality, a combination that continues to be consistently supported by the available clinical evidence, as consistently demonstrated in recent systematic reviews and meta-analyses [35–37].

This interpretation is consistent with recent systematic reviews and meta-analyses, which have uniformly demonstrated favourable clinical outcomes for calcium-silicate biomaterials across a range of VPT procedures and reinforced their position as the current clinical benchmark. These reviews have consistently demonstrated favourable clinical outcomes across different treatment approaches and biomaterial comparisons while highlighting the continued importance of biologically based, minimally invasive management strategies [35–37]. Importantly, whereas these reviews primarily evaluate the clinical performance of established biomaterials, the present review additionally considers scaffold-based regenerative biomaterials within a translational framework, thereby extending current understanding beyond comparative clinical outcomes alone [35–37].

The findings of the present review suggest that the principal barriers to clinical translation extend beyond demonstration of biological feasibility. Although scaffold-based regenerative biomaterials consistently promoted regenerative processes under experimental conditions, the available evidence remains largely restricted to short-term preclinical investigations conducted under controlled laboratory conditions [20–24]. Consequently, important questions relating to long-term performance, reproducibility, and clinical implementation remain unanswered. In contrast, calcium-silicate biomaterials are supported by a substantial body of human clinical evidence demonstrating predictable outcomes under routine clinical conditions [35–37]. This contrast illustrates that successful translation requires not only biological innovation, but also robust clinical evidence capable of demonstrating reproducible patient benefit.

The methodological limitations of the available evidence should be considered when interpreting the findings of the present review. Nevertheless, these limitations largely reflect the current stage of regenerative endodontic research rather than shortcomings unique to the present analysis. By synthesising preclinical and clinical evidence within a single translational framework, this review provides a contemporary perspective on how biological innovation is progressing towards clinical application. Rather than attempting to establish comparative superiority between scaffold-based regenerative biomaterials and calcium-silicate biomaterials, the review identifies where the current evidence is strongest, where important uncertainties remain, and where future translational efforts should be directed. In doing so, it provides a framework for interpreting emerging regenerative technologies within the broader context of contemporary VPT.

Beyond the individual findings of the included studies, the present review offers an opportunity to reframe how regenerative endodontics is interpreted. Rather than viewing scaffold-based regenerative biomaterials and calcium-silicate biomaterials as competing technologies, the available evidence suggests that they currently represent different stages along the same translational pathway. Consequently, the future of regenerative endodontics should not be viewed simply as the pursuit of increasingly sophisticated biomaterials, but as the pursuit of clinically meaningful regeneration supported by robust translational evidence. This distinction is important because biological innovation and clinical innovation are not synonymous. Progress within the field will ultimately be determined not only by advances in biomaterial engineering, but also by the ability to demonstrate reproducible improvements in clinically relevant outcomes under routine clinical conditions.

The findings of the present review also provide a clear direction for future research. The immediate priority is no longer to demonstrate that scaffold-based regenerative biomaterials can stimulate biological regeneration under experimental conditions, but to establish whether these biological advantages translate into reproducible improvements in clinically relevant outcomes. Achieving this objective will require well-designed human clinical studies directly comparing scaffold-based regenerative biomaterials with calcium-silicate biomaterials using standardised outcome measures and sufficiently long follow-up periods. Future investigations should also incorporate clinically relevant models that better reflect the biological complexity of VPT, including microbial challenge, patient-related variability, and functional assessment of regenerated tissue. Addressing these priorities will strengthen the translational evidence base and help determine whether scaffold-based regenerative biomaterials can deliver clinically meaningful advantages beyond those currently achieved with calcium-silicate biomaterials.

Regenerative endodontics stands at an important stage in its evolution. The evidence synthesised in the present review suggests that the future of the field will depend not only upon continued biological innovation, but also upon understanding how that innovation can be translated into reproducible and clinically meaningful benefit. Calcium-silicate biomaterials remain the current benchmark for predictable VPT, while scaffold-based regenerative biomaterials continue to expand the biological possibilities of what may ultimately become achievable [35–37]. Whether these two paths will ultimately converge, or whether future advances will redefine the boundaries of pulp regeneration, remains one of the most compelling questions facing contemporary endodontics. The answer is unlikely to be determined by biological sophistication alone, but by our ability to understand when biological innovation truly becomes clinical innovation.

## Conclusion

Current evidence supports calcium-silicate biomaterials as the clinical benchmark for VPT, while scaffold-based regenerative biomaterials demonstrate considerable biological promise that has yet to be translated into predictable clinical benefit. Rather than diminishing the significance of regenerative research, this distinction highlights the principal challenge facing contemporary regenerative endodontics: transforming biological innovation into reproducible improvements in patient care. Ultimately, the future of the field will be determined not only by what can be achieved under experimental conditions, but by what can be delivered consistently and meaningfully in clinical practice. Whether future regenerative strategies will ultimately redefine the boundaries of VPT remains an open question, one that will be answered not by biological innovation alone, but by the strength of the clinical evidence that follows.

## Supplementary information

Supplementary Material 1 - Electronic Search Strategy

Supplementary Material 2 - PRISMA Checklist

## Data Availability

All data analysed during this study are included in the published article. No new datasets were generated.

## References

[1] Galler KM, Weber M, Korkmaz Y, Widbiller M, Feuerer M. Inflammatory response mechanisms of the dentine–pulp complex and the periapical tissues. Int J Mol Sci. 2021;22:1480. 10.3390/ijms22031480 PMC7867227 33540711

[2] Ricucci D, Loghin S, Siqueira JF. Correlation between clinical and histologic pulp diagnoses. J Endod. 2014;40:1932–9. 10.1016/j.joen.2014.08.010 25312886

[3] Simon S, Perard M, Zanini M, Smith AJ, Charpentier E, Djole SX, et al. Should pulp chamber pulpotomy be seen as a permanent treatment? Some preliminary thoughts. Int Endod J. 2013;46:79–87. 10.1111/j.1365-2591.2012.02113.x 22900881

[4] Taha NA, Khazali MA. Partial pulpotomy in mature permanent teeth with clinical signs indicative of irreversible pulpitis: a randomised clinical trial. J Endod. 2017;43:1417–21. 10.1016/j.joen.2017.03.033 28673494

[5] Aguilar P, Linsuwanont P. Vital pulp therapy in vital permanent teeth with cariously exposed pulp: a systematic review. J Endod. 2011;37:581–7. 10.1016/j.joen.2010.12.004 21496652

[6] Hilton TJ, Ferracane JL. Pulp capping with calcium hydroxide and mineral trioxide aggregate: comparison of success rates. Oper Dent. 2010;35:431–6.

[7] Bjørndal L, Simon S, Tomson PL, Duncan HF. Management of deep caries and the exposed pulp. Int Endod J. 2019;52:949–73. 10.1111/iej.13128 30985944

[8] Duncan HF, Galler KM, Tomson PL, Simon S, El-Karim IA, Kundzina R, et al. European Society of Endodontology position statement: management of deep caries and the exposed pulp. Int Endod J. 2019;52:923–34. 10.1111/iej.13080 30664240

[9] Parirokh M, Torabinejad M. Mineral trioxide aggregate: a comprehensive literature review—Part I: chemical, physical and antibacterial properties. J Endod. 2010;36:16–27. 10.1016/j.joen.2009.09.006 20003930

[10] Camilleri J. Biodentine: a dentine substitute and a bioactive material. J Dent. 2013;41:613–20. 10.1016/j.jdent.2013.05.003 23685034

[11] Galler KM, D’Souza RN, Federlin M, Cavender AC, Hartgerink JD, Hecker S, et al. Dentin conditioning codetermines cell fate in regenerative endodontics. J Endod. 2011;37:1536–41. 10.1016/j.joen.2011.08.027 22000458

[12] Nakashima M, Iohara K. Regeneration of dental pulp by stem cells. Adv Dent Res. 2011;23:313–9. 10.1177/0022034511405323 21677085

[13] Rosa V, Zhang Z, Grande RH, Nör JE. Dental pulp tissue engineering in full-length human root canals. J Dent Res. 2013;92:970–5. 10.1177/0022034513505772 PMC3797540 24056227

[14] Murray PE, Garcia-Godoy F, Hargreaves KM. Regenerative endodontics: a review of current status and a call for action. J Endod. 2007;33:377–90. 10.1016/j.joen.2006.09.013 17368324

[15] Hargreaves KM, Diogenes A, Teixeira FB. Treatment options: biological basis of regenerative endodontic procedures. J Endod. 2013;39:S30–S43. 10.1016/j.joen.2012.11.025 PMC3589799 23439043

[16] Schmalz G, Widbiller M, Galler KM. Signaling molecules and pulp regeneration. J Endod. 2017;43:S7–S11. 10.1016/j.joen.2017.06.003 28844306

[17] Widbiller M, Schmalz G. Endodontic regeneration: hard tissue formation and tissue engineering approaches. J Endod. 2015;41:S27–S36.

[18] Huang GT. Pulp and dentin tissue engineering and regeneration: current progress. J Dent Res. 2009;88:639–47. 10.2217/rme.09.45 PMC2785543 19761395

[19] Diogenes A, Ruparel NB, Shiloah Y, Hargreaves KM. Regenerative endodontics: a way forward. J Am Dent Assoc. 2016;147:372–80. 10.1016/j.adaj.2016.01.009 27017182

[20] Cunha RS, Sinjari B, De-Deus G, et al. Regenerative potential of 3D-printed GelMA microgels combined with dentine matrix proteins in vital pulp therapy: an experimental study. J Endod. 2023;49:1012–20.

[21] Han L, Zhang L, Chen X, Li Y, Wang Y, Liu H, et al. Evaluation of GelMA/β-TCP nanocomposite scaffolds for pulp tissue regeneration in a rat molar model. Acta Biomater. 2024;170:245–56.

[22] Tao X, Chen Y, Zhang J, Li M, Wang X, Liu Z, et al. Injectable bioceramic hydrogel scaffolds promote neurovascular regeneration in vital pulp therapy. Bioact Mater. 2025;32:112–23.

[23] Yi J, Wang Z, Li Q, Chen Y, Zhang X, Liu Y, et al. Hierarchical hydrogel scaffolds for dentine–pulp complex regeneration in a rat model. Biomaterials. 2024;301:122257.

[24] Zhang Y, Liu H, Wang X, Chen Z, Li J, Zhao Y, et al. Nanocomposite scaffolds for regeneration of the dentine–pulp complex: an in vivo rabbit study. Dent Mater. 2024;40:365–74.

[25] Page MJ, McKenzie JE, Bossuyt PM, Boutron I, Hoffmann TC, Mulrow CD, et al. The PRISMA 2020 statement: an updated guideline for reporting systematic reviews. BMJ. 2021;372:n71. 10.1136/bmj.n71 PMC8005924 33782057

[26] Joanna Briggs Institute. JBI Manual for Evidence Synthesis. Adelaide: Joanna Briggs Institute; 2020.

[27] Hooijmans CR, Rovers MM, de Vries RB, Leenaars M, Ritskes-Hoitinga M, Langendam MW. SYRCLE’s risk of bias tool for animal studies. BMC Med Res Methodol. 2014;14:43. 10.1186/1471-2288-14-43 PMC4230647 24667063

[28] Murad MH, Mustafa RA, Schünemann HJ, Sultan S, Santesso N. Rating the certainty in evidence in the absence of a single estimate of effect. Evid Based Med. 2017;22:85–87. 10.1136/ebmed-2017-110668 PMC5502230 28320705

[29] Aguilar P, Linsuwanont P, Taha NA, et al. Randomised clinical trial comparing mineral trioxide aggregate and Biodentine in vital pulp therapy of permanent teeth. Int Endod J. 2021;54:1125–34.

[30] Silva EJNL, Rover G, Belladonna FG, et al. Clinical evaluation of Biodentine pulpotomy compared with root canal treatment in mature permanent teeth: a randomised clinical trial. J Endod. 2025;51:215–23.

[31] Taha NA, Abdelkhader SZ, Khazali MA, et al. Outcomes of full pulpotomy using bioceramic putty versus root canal treatment in mature permanent teeth: a randomised clinical trial with 24-month follow-up. Int Endod J. 2023;56:684–93.

[32] Zhu Q, Li R, Peng B, et al. Clinical outcomes of vital pulp therapy using iRoot BP Plus compared with root canal treatment: a prospective cohort study. Clin Oral Investig. 2024;28:1221–30.

[33] Baranwal AK, Meena N, Yadav S, et al. Randomised clinical trial comparing Biodentine and mineral trioxide aggregate for pulpotomy in permanent teeth. J Endod. 2025;51:410–8.

[34] Gilhotra JS, Gill GS, Kaur A, et al. Comparative clinical evaluation of Biodentine, MTA, Bio-C Repair and EndoSequence BC-RRM in vital pulp therapy: a randomised controlled trial. Clin Oral Investig. 2024;28:4875–84.

[35] Asgary S, Shamszadeh S, Shirvani A, et al. Management strategies for pulpitis in vital permanent teeth in children and adolescents: a systematic review and meta-analysis of randomized clinical trials. Eur Arch Paediatr Dent. 2025;26:451–63. 10.1007/s40368-025-01021-w 40117109

[36] Komora P, Vámos O, Gede N, et al. Comparison of bioactive material failure rates in vital pulp treatment of permanent matured teeth: a systematic review and network meta-analysis. Sci Rep. 2024;14:18421. 10.1038/s41598-024-69367-7 PMC11310317 39117767

[37] Lamb AR, Ghivari S, Meshram R, et al. Comparative evaluation of calcium-enriched mixture and mineral trioxide aggregate in vital pulp therapy of molars: a systematic review. J Conserv Dent Endod. 2026;29:127–34. 10.4103/JCDE.JCDE_882_25 PMC12935437 41756790

