# Supplementary material for: Scaffold-based and calcium-silicate biomaterials in vital pulp therapy: a systematic review of current evidence and translational perspectives
Source: BDJ Open. 2026 Oct 31;12:103. doi: 10.1038/s41405-026-00480-7 (PMC13723456; doi:10.1038/s41405-026-00480-7)
Supplement: Supplementary file 1 [file 41405_2026_480_MOESM1_ESM.docx]

**Electronic Search Strategy**

**Database: PubMed (MEDLINE)**

**Search conducted:** March 2025

("vital pulp therapy" OR "pulpotomy" OR "direct pulp capping")
AND
("scaffold" OR "hydrogel" OR "3D printed scaffold" OR "tissue engineering")
AND
("calcium silicate" OR "mineral trioxide aggregate" OR "Biodentine")

**Search Limits**

- Language: English
- Study types: Clinical studies and in vivo animal studies
- Timeframe: From database inception to March 2025

**Additional Search Methods**

- Manual screening of reference lists of included studies
- Cross-referencing of relevant review articles

**Notes**

Search strategies were adapted appropriately for Scopus and Web of Science using equivalent keywords and Boolean operators.
