# Supplementary material for: Scaffold-based and calcium-silicate biomaterials in vital pulp therapy: a systematic review of current evidence and translational perspectives
Source: BDJ Open. 2026 Oct 31;12:103. doi: 10.1038/s41405-026-00480-7 (PMC13723456; doi:10.1038/s41405-026-00480-7)
Supplement: Supplementary file 2 [file 41405_2026_480_MOESM2_ESM.docx]

**PRISMA CHECKLIST**

| **Section** | **Item** | **Description** | **Location in Manuscript** |
| --- | --- | --- | --- |
| Title | 1 | Identify the report as a systematic review | Title |
| Abstract | 2 | Provide a structured summary including background, methods, results and conclusions | Abstract |
| Introduction | 3 | Describe the rationale for the review in the context of existing knowledge | Introduction |
| Introduction | 4 | State the objectives or research question | Introduction |
| Methods | 5 | Specify eligibility criteria for included studies | Methods |
| Methods | 6 | Specify information sources (databases searched) | Methods |
| Methods | 7 | Present the full search strategy | Methods |
| Methods | 8 | Describe the study selection process | Methods |
| Methods | 9 | Describe the data collection process | Methods |
| Methods | 10 | List and define data items extracted from studies | Methods |
| Methods | 11 | Describe methods used to assess risk of bias | Methods |
| Methods | 12 | Describe effect measures used | Not applicable (narrative synthesis) |
| Methods | 13 | Describe methods of data synthesis | Methods |
| Methods | 14 | Describe assessment of reporting bias | Not applicable |
| Methods | 15 | Describe certainty assessment | Methods |
| Results | 16 | Report the results of the study selection process | Results / Figure 1 (PRISMA diagram) |
| Results | 17 | Cite included studies and describe their characteristics | Results / Table 1 |
| Results | 18 | Present risk of bias assessments | Results |
| Results | 19 | Present results of individual studies | Results |
| Results | 20 | Present results of syntheses | Results |
| Results | 21 | Present reporting bias assessment | Not applicable |
| Results | 22 | Present certainty of evidence | Results |
| Discussion | 23 | Provide interpretation of results in context of existing evidence | Discussion |
| Discussion | 24 | Discuss limitations of evidence | Discussion |
| Discussion | 25 | Discuss limitations of the review process | Discussion |
| Other information | 26 | Provide funding sources | Declarations |
| Other information | 27 | Declare competing interests | Declarations |
| Other information | 28 | Provide data availability information | Declarations |
